# Age effects on EEG correlates of the Wisconsin Card Sorting Test

**DOI:** 10.14814/phy2.12390

**Published:** 2015-07-26

**Authors:** Nuno S Dias, Daniela Ferreira, Joana Reis, Luís R Jacinto, Luís Fernandes, Francisco Pinho, Joana Festa, Mariana Pereira, Nuno Afonso, Nadine C Santos, João J Cerqueira, Nuno Sousa

**Affiliations:** 1 Life and Health Science Research Institute (ICVS), School of Health Sciences, University of MinhoBraga, Portugal; 2 ICVS/3B’s - PT Government Associate LaboratoryBraga, Guimarães, Portugal; 3 Clinical Academic Center-BragaBraga, Portugal; 4 DIGARC, Polytechnic Institute of Cávado and AveBarcelos, Portugal; 5 Department of Industrial Electronics, School of Engineering, University of MinhoBraga, Portugal

**Keywords:** Aging, cognition, EEG rhythms

## Abstract

Body and brain undergo several changes with aging. One of the domains in which these changes are more remarkable relates with cognitive performance. In the present work, electroencephalogram (EEG) markers (power spectral density and spectral coherence) of age-related cognitive decline were sought whilst the subjects performed the Wisconsin Card Sorting Test (WCST). Considering the expected age-related cognitive deficits, WCST was applied to young, mid-age and elderly participants, and the theta and alpha frequency bands were analyzed. From the results herein presented, higher theta and alpha power were found to be associated with a good performance in the WCST of younger subjects. Additionally, higher theta and alpha coherence were also associated with good performance and were shown to decline with age and a decrease in alpha peak frequency seems to be associated with aging. Additionally, inter-hemispheric long-range coherences and parietal theta power were identified as age-independent EEG correlates of cognitive performance. In summary, these data reveals age-dependent as well as age-independent EEG correlates of cognitive performance that contribute to the understanding of brain aging and related cognitive deficits.

## Introduction

In an increasingly aged society, it is critical to understand how the brain ages and develops strategies to reverse or decelerate unhealthy aging. This knowledge may provide strategies to prevent, ameliorate or at least delay cognitive impairments that entail high personal, social and financial costs (Lustig et al. 2009). Decrease in neuronal plasticity and connectivity may be signatures of cognitive decline, from healthy aging to dementia. In fact, the progressive loss of plasticity associated with increasing age leads to a decrease in the retention of parallel information, information processing, and short-term memory retention (Rossini et al. 2007). Some aging theories also show decrements in memory control processes as one of the key determinants of cognitive decline (Werkle-Bergner et al. 2012).

The electroencephalogram (EEG) has been used to determine which neuronal areas, their function and rhythms, are altered with aging (Rossini et al. 2007). The identification of age-related EEG phenotypes is challenging as the aging process is complex and heterogeneous. Yet, previous EEG studies have highlighted some correlates of cognitive performance. More specifically, studies on working and episodic memory have revealed an increase in theta activity during the encoding phase (Klimesch 1999; Head et al. 2009; Itthipuripat et al. 2013); importantly theta synchronization is often associated with good performance during cognitive tasks (Klimesch 1996, 1999). Alpha activity has also been associated with cognitive performance through its role on attention and binding processes (Klimesch 1999; Herrmann and Knight 2001). Many studies suggest that increases in alpha power are closely related to the successful inhibition of irrelevant information (Herrmann and Knight 2001; Werkle-Bergner et al. 2012). Alpha synchronization, on the other hand, provides a mechanism for the timing of neuronal information processing and the successful formation of integrated representations (Klimesch 1999; Werkle-Bergner et al. 2012).

Wisconsin Card Sorting Test (WCST) requires the subject to match different cards according to their symbols’ color, quantity and shapes, and is often applied in EEG cognitive studies (Barceló 1999). The WCST is able to measure cognitive flexibility, that is the ability to alter a behavioral response mode in the face of changing contingencies (i.e., rule shifting), and working memory, which is the ability to manipulate data in short-term periods. In the first studies, the WCST was used mainly to investigate the frontal lobe, but more recently it has been shown as a powerful task to study other anterior and posterior brain regions (Barceló 2001; González-Hernández et al. 2002).

In this study, we sought to understand the age effects on EEG correlates of performance during the WCST and thus, discriminate EEG markers of brain alterations naturally occurring with aging from those hypothetically indicative of age-independent cognitive deficits. Considering the expected decline of cognitive performance with increasing age, young, midage, and elder subjects performed the WCST while their EEG signals were acquired. Analysis of variance and linear regression analysis were applied in order to identify the linear dependence of each EEG marker from the independent variables: age and performance scores. The partial correlations between EEG features and the independent variables allowed us to identify age-dependent and age-independent neuronal markers.

## Methods

### Ethical approval

The study was conducted in accordance with the Declaration of Helsinki (59th Amendment), and was approved by national and local ethics committees. Potential participants were explained the study goals and the neurocognitive assessment.

### Subjects

The 62 participants of this study were divided into three age groups:

- Young group: 19 subjects aged between 20- and 34-years old (24.3 ± 3.1 SD); 10 females and 9 males;
- Mid-age group: 28 subjects aged between 51- and 64-years old (56.4 ± 3.7 SD); 11 females and 17 males;
- Elderly group: 15 subjects aged between 67- and 82-years old (average: 73.5; standard deviation: 5.6); 6 females and 9 males.

Midage and elderly participants were selected from the Guimarães and Vizela local area health authority registries. The sample is part of a larger cohort (*n* = 3038, males and females 18–97 years of age, from an original n = 4000; drop-out rate for the age group over 50 years of age: 27.8%) (Santos et al. 2013). The young subjects were recruited from the research and student communities of University of Minho. The primary exclusion criteria included inability to understand informed consent, participant choice to withdraw from the study, dementia and/or diagnosed neuropsychiatric and/or neurodegenerative disorder (medical records). The Edinburgh Handedness Test was used to determine laterality. All subjects were right-handed and had normal or corrected-to-normal vision. All subjects answered a questionnaire about their educational, medical (including medication), and family records and voluntarily signed the informed consent to participate on these experiments.

### Electroencephalogram acquistion

**Figure 1:**
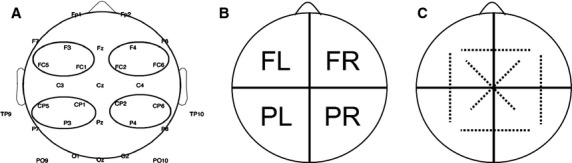
(A) Electrodes arrangement used in EEG recording and electrode locations selected for four electrode pools; (B) electrode pools considered for power analysis: FL-frontal left; FR-frontal right; PL-parietal left; PR-parietal right; and (C) pool couplings considered for coherence analysis: FL-FR, FL-PL, FL-PR, FR-PR, FR-PL, and PL-PR.

Electroencephalogram (EEG) signals were recorded with a Quickamp (Brain Products, GmbH) using the 10–20 system (32 electrodes plus reference and ground electrodes), as represented in Fig.1A. The EEG system consisted of Ag/AgCl active electrodes, an actiCAP® (Brain Products, GmbH) for the placement of the 32 electrodes, electrolyte gel to decrease the contact impedance between electrodes and the scalp and straps to keep the cap in place. The OpenVibe (http://openvibe.inria.fr/) software was used to acquire and synchronize the EEG signals with the WCST paradigm and to save data for offline analysis (Renard et al. 2010). A version of the WCST which has been implemented with the Psychology Experiment Building Language (PEBL - http://pebl.sourceforge.net/) was applied in this study (Mueller and Piper 2014). Figure1 presents the electrode locations of the acquired EEG signals.

The subjects were seated, in an illuminated and acclimatized room, distancing 50–70 cm from the computer screen, with touch technology. All subjects were asked to answer as quickly as possible, always with the right hand and not make any movements beyond those required. Each subject performed three sessions of the WCST, while the EEG signals were recorded with a sampling rate of 1024 samples per second. Before each WCST, a 30 sec baseline was recorded where the subjects were looking at the computer’s monitor with a black screen as relaxed as possible.

### Wisconsin Card Sorting Test

The WCST is constituted by four decks of cards that differ according to three categories: colors (red, green yellow, and blue), shapes (triangle, star, cross, and circle) and number of symbols (from 1 to 4). When a card appears at the bottom of the screen, the subject has to match it to one of the four decks, following one of the three categories. The subject has to touch in the deck that matches the card according to the category in use. After each answer, the feedback (‘correct’ or ‘incorrect’) is given to the subject. Once the subject discovers the category (e.g., symbol, color, or number), he/she should follow it until change, which occurs after 10 correct card matches. When the category in use changes, the subject has to discover the new category, which is always different from the previous one. The test ends after nine completed categories or when a total of 128 cards are drawn.

The performance measures considered on the WCST are as follows: completed categories, perseverative errors and nonperseverative errors. The number of completed categories indicates the number of categories in which the subject receives ten times the feedback ‘correct’. The perseverative errors occur when the subject continues to answer according to the category used before rule change. The nonperseverative errors are a set of two types of errors: efficient and distraction. The efficient errors are related to the strategy of discovering a new category, when the subject first receives a negative feedback after 10 correct card matches. The distracting errors occur when the subject selects a card incorrectly after discovering the category in use (Barceló 1999). Each performance measure (i.e., completed categories, perseverative errors and nonperseverative errors) was standardized according to the eq. 1:

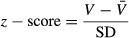
1

where *V* is the result of each subject in a performance measure; 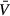 and SD are the subject mean and standard deviation for each performance measure.

The performance *z*-score is a weighted average of the completed categories, perseverative errors and non-perseverative errors *z*-scores. The perseverative and non-perseverative errors have a negative weight and the completed categories have a positive weight on the *z*-score pooling. Good performers should maximize the number of completed categories and minimize perseverative and non-perseverative errors.

### Clustering analysis – performance vs. age

**Figure 2:**
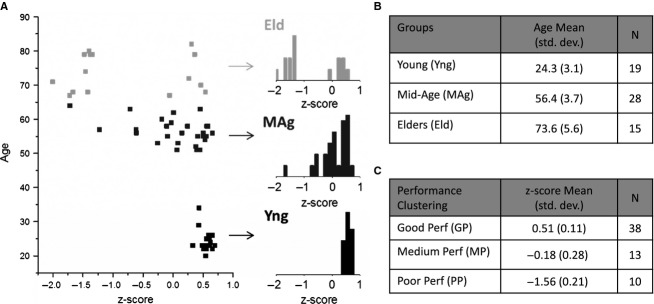
(A) Scatter plot of all subjects included in the study according to age and *z*-score performance on WCST; inset histograms show an increasing tendency to a bimodal *z*-score distribution as age increases; (B) statistics for young, mid-age and elders groups; (C) statistics for good performers, medium performers and poor performers clusters.

The scattering of the subjects according to *z*-score and age is represented in Fig.2A. Although cognitive performance has been described to decrease with age (Lustig et al. 2009), as expected, some midage and even elderly subjects performed the WCST as well as the young subjects. Thus, while the young group presents a unimodal distribution of *z*-scores, the midage and elders groups seem to present bimodal distributions on *z*-scores. EEG power spectral density and spectral coherence were investigated in the participants in respect to their age and performance success at the WCST. Using K-means clustering analysis, three performance levels were defined in respect to the mean *z*-score: good performers (*n* = 38; 0.51 ± 0.11 SD), medium performers (*n* = 13; −0.18 ± 0.28 SD), poor performers (*n* = 10; −1.56 ± 0.21 SD).

### Signal preprocessing

Analyzer 2 (Brain Products, GmbH) was used to analyze the EEG signals. Signals were filtered with: a Butterworth zero-phase high-pass filter (cutoff frequency of 0.3 Hz, time constant of 0.5305 sec, and slope of 48 db/Oct), a Butterworth zero-phase low-pass filter (cutoff frequency of 100 Hz and slope of 48 db/Oct) and a Butterworth zero-phase notch filter rejecting the frequency of 50 Hz. After filtering the signals, the ocular artifacts were corrected using an algorithm based on independent component analysis (Jung et al. 2000). In order to increase the independence of the signals between neighboring electrode locations, an implementation of the current source density (CSD) method was applied on the filtered data (Perrin et al. 1989). CSD reduces the redundancy, ambiguity, and reference-dependency of volume-conducted EEG measures (Tenke and Kayser 2012).

The segmentation of EEG signals in 5 sec epochs was applied on the whole data set, from the appearance of the first card to the last feedback message, as well as for the 30 sec baseline period. The EEG data were not segmented in respect to any task event. The power spectral density and the spectral coherence were calculated for each 5 sec segment, on theta (approx. 4–8 Hz) and alpha (approx. 8–13 Hz) frequency bands. The artifacts still remaining in the data (possibly due to subject movement or poor electrode contact) were removed by rejecting the segments with current source density amplitude higher than 500 *μ*V/m^2^ and also with a difference between the maximum and minimum values higher than 800 *μ*V/m^2^.

From the 32 electrodes recorded (Fig.1A), only 12 electrodes, were used to calculate the power spectral density and the spectral coherence for theta and alpha rhythms. Four electrode pools were selected for analysis according to Fig.1B: frontal region on left hemisphere (FL), frontal region on right hemisphere (FR), parietal region on left hemisphere (PL) and parietal region on right hemisphere (PR). The border electrodes between regions were discarded. As a result of channel pooling, the average of three channels per area were considered for power spectral density and coherence analyses (Fig.1B and C): (1) FL pool: FC5, F3 and FC1 electrodes; (2) FR pool: FC6, F4 and FC2 electrodes; (3) PL pool: CP5, P3 and CP1 electrodes; (4) PR pool: CP6, P4 and CP2 electrodes. The frequency limits of each frequency band were adjusted according to the alpha peak of the subject. The alpha peak is the value of the frequency for which the amplitude of the signal is higher in the frequency band of 8–13 Hz. The alpha band was adjusted in ± 2 Hz from the value of the alpha peak and the theta band was adjusted between alpha peak minus 7 Hz and alpha peak minus 3 Hz. Lateral asymmetry of the alpha peak was calculated as the difference between FL and FR or between PL and PR alpha peak frequencies.

### Power spectral density analysis

The power spectral density (PSD) was calculated for the 5 sec data segments, during the baseline period and during the task period – execution of WCST, through absolute or baseline-corrected measures using the Fourier transform. The power values for alpha and theta frequency bands were extracted from the PSD averaged over all 5 sec segments.

### Spectral coherence analysis

The coherence of the EEG signals from two electrodes is a measure of the degree of association between the spectra of the two channels, providing possible information about the functional coupling between two neuronal areas. Mathematically, coherence is defined as the normalized cross frequency spectrum between two EEG signals recorded from different locations of the scalp (Sanei and Chambers 2007). The coherence was calculated using:

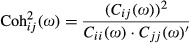
2where 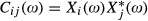 is the cross-correlation coefficient between the Fourier transform of the EEG signals of channel *i* and channel *j* and *C*_ii_ (*ω*) is the autocorrelation of the Fourier transform of the EEG signal from *i* channel (Sanei and Chambers 2007). Absolute and baseline-corrected measures were calculated. Figure1C presents the color scheme for the coherence between the six region pairs under study.

### Statistical analysis

The effects of the age and performance factors on power spectral density and spectral coherence of alpha and theta rhythms were analyzed through a two-way ANOVA. Linear regression analyses were also applied to assess the dependence of each EEG marker from the independent variables: age and performance *z*-scores. Considering that age and performance are highly correlated factors (Pearson coefficient: −0.621; *P*-value < 0.01), the partial correlations between each EEG marker and age or between each EEG marker and performance *z*-scores were also assessed. Additionally, age effects on PSD and coherence were analyzed on a sub-group of subjects with best performance on WCST and performance effects on PSD and coherence were studied on a sub-group consisting of the oldest subjects. The statistical analyses were performed using Matlab® (Mathworks, Natick, MA) and IBM SPSS Statistics v.22 (IBM, New York, NY) and plots were generated from Origin® (OriginLab, Northampton, MA).

## Results

### EEG baseline recordings

**Figure 3:**
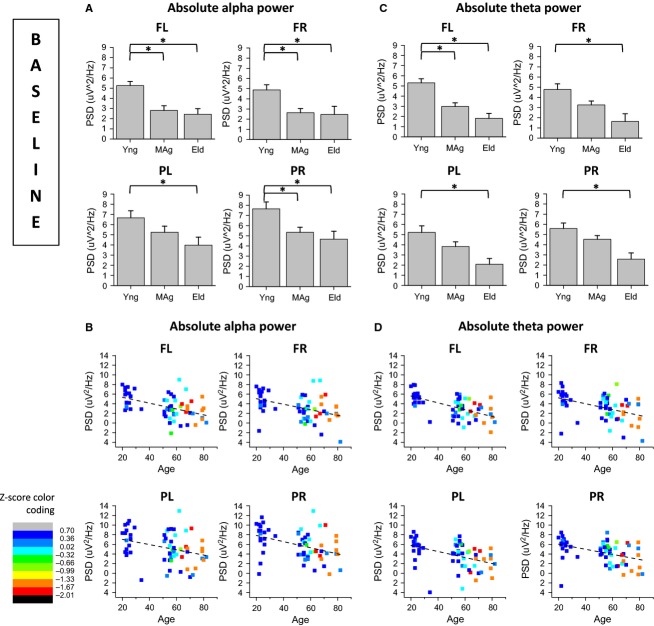
Analysis of age effects on Power Spectral Density (PSD) of alpha and theta rhythms during baseline recordings; (A) alpha PSD is generally higher on young subjects than on middle-age and elders; (B) alpha PSD is inversely correlated with age in all four electrode pools; (C) theta PSD is higher on young rather than on elder subjects in all electrode pools, and higher than on middle-aged subjects on FL pool; (D) theta PSD is inversely correlated with age in all four electrode pools.

Figures3A and C present the age effects on power spectral density of alpha and theta rhythms, respectively, for frontal left (FL), frontal right (FR), parietal left (PL), and parietal right (PR) scalp regions, during baseline recordings. Figures3B and D, present the correlations between alpha power and age and between theta power and age, respectively.

The basal alpha power of young subjects was higher than that of older subjects’ on FL, FR, PL and PR electrode pools (Fig.3A). Basal alpha power of young subjects was also higher than midage subjects’ on FL, FR and PR electrode pools (Fig.3A). Basal alpha power was inversely correlated with age (Fig.3C), controlling for the partial correlation between age and performance *z*-scores, on FL (partial correlation = −0.43), FR (partial correlation = −0.37), PL (partial correlation = −0.30) and PR (partial correlation = −0.39). Basal theta power of young subjects was higher than elders’ on FL, FR, PL and PR electrode pools (Fig.3B). Basal alpha power of young subjects was also higher than midage subjects’ on FL electrode pool (Fig.3B). Basal theta power was also inversely correlated with age (Fig.3D), controlling for the partial correlation between age and performance *z*-scores, on FL (partial correlation = −0.56), FR (partial correlation = −0.43), PL (partial correlation = −0.37) and PR (partial correlation = −0.33). There were no significant effects of age on spectral coherence of EEG during baseline recordings. The inverse correlation between alpha and theta power during baseline and age was confirmed through a linear regression analysis design (see Tables S1.3 and S1.4, in Appendix S1). Detailed statistical results of two-way ANOVA are also presented on Tables S1.1 and S1.2 in Appendix S1.

### EEG recordings during the WCST

#### Alpha and theta power recorded during the WCST is generally inversely correlated with age

**Figure 4:**
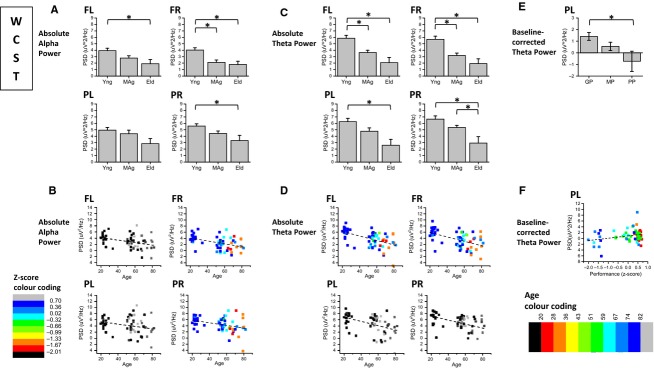
Analysis of Power Spectral Density (PSD) of alpha and theta rhythms with simultaneous WCST performance; (A) alpha PSD is generally higher on young subjects than on middle-age and elders, during WCST performance; (B) alpha PSD is inversely correlated with age in right hemisphere; (C) theta PSD is generally higher on young subjects rather than on elders and middle-age subjects; (D) theta PSD is inversely correlated with age in frontal regions; (E) Baseline-corrected theta power recorded simultaneously to WCST is higher for good than for poor performers on PL scalp region; (F) Baseline-corrected theta power on PL is directly correlated with performance success on WCST.

Figure4A and C present the age effects on absolute power spectral density of alpha and theta rhythms, respectively, for FL, FR, PL, and PR scalp regions, recorded during the performance of the WCST. Figure4B and D, present the correlations between alpha power and age and between theta power and age, respectively, for the same task. Figure4E presents baseline-corrected PL theta power recorded simultaneously to WCST performance and Fig.4F shows the correlation between corrected theta power and performance *z*-score.

During the performance of the WCST, alpha power of young subjects was higher than elders’ for FL, FR and PR electrode pools and higher than midage subjects for FR pool, as shown in Fig.4A. Alpha power was inversely correlated with age on FR (partial correlation = −0.31) and PR (partial correlation = −0.29) electrode pools (Fig.4B). Theta power of young subjects was higher than elders’ on FL, FR, PL, and PR, and higher than midage subjects on FL and FR pools (Fig.4C). Mid-age subjects presented higher theta power than elders on PR electrode pool. The power of theta rhythm acquired during the WCST was inversely correlated with age on FL (partial correlation = −0.43) and FR pools (partial correlation = −0.41), according to Fig.4D. While the alpha power was generally higher during the resting state (baseline) than in the activity state (WCST performance) across subjects, the opposite was generally valid for theta power. Linear regression analysis on Tables S2.3. and S2.4 in Appendix S2, confirms the inverse correlation between alpha power and age on the right hemisphere and the inverse correlation between theta power and age on frontal sites (see supplementary data). Detailed statistical results of two-way ANOVA are presented on Tables S2.1 and S2.2 in Appendix S2.

As shown in Fig.4E, baseline-corrected theta power recorded during the WCST was higher for good than for poor performers on left parietal scalp region. Baseline-corrected theta power was directly correlated (partial correlation = 0.31) with performance success on WCST, as shown in Fig.4F (see detailed statistical results on Tables S4.1. and S4.2., in Appendix S4).

#### Interhemispheric and fronto-parietal coupling between frontal right and other scalp regions is inversely correlated with age, for alpha and theta rhythms recorded during WCST performance

**Figure 5:**
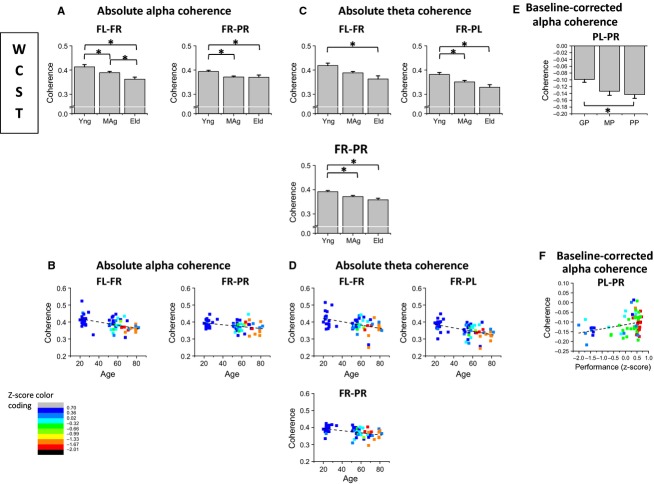
Analysis of Spectal Coherence of alpha and theta rhythms acquired simultaneously to WCST performance; (A) alpha coherence is higher for young rather than older subjects on FL-FR and FR-PR scalp regions; (B) alpha spectral coherence is inversely correlated with age both on FL-FR and FR-PR couplings; (C) theta coherence is higher for young rather than older subjects on frontal inter-hemispheric FL-FR, FR-PL and FR-PR couplings; (D) theta spectral coherence is inversely correlated with age on FL-FR, FR-PL and FR-PR couplings; (E) baseline-corrected PL-PR alpha coherence is higher for good than for poor performers; (F) baseline-corrected PL-PR alpha coherence is directly correlated with performance success on WCST.

Figure5A and C present the age effects on absolute spectral coherence of alpha and theta rhythms, respectively, for coherence between FL and FR 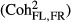, coherence between FR and PR 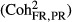 and coherence between FR and PL 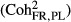, recorded during the WCST. Figures5B and D, present the correlations between alpha coherence and age and between theta coherence and age, respectively. Figure5E presents the performance effects on baseline-corrected alpha coherence between PL and PR 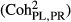 and Fig.5F shows the correlation between baseline-corrected alpha coherence and performance *z*-scores.

Absolute alpha spectral coherence recorded during the WCST was higher for young subjects on frontal interhemispheric 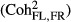 and right fronto-parietal 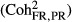 scalp regions (Fig.5A). Absolute alpha spectral coherence was inversely correlated with age, controlling for the partial correlation between age and performance *z*-scores, both on 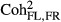 (partial correlation = −0.29) and 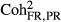 (partial correlation = −0.27) coupling (Fig.5B). Absolute theta coherence was higher for young rather than elder subjects on frontal interhemispheric 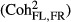, interhemispheric fronto-parietal 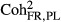 and right fronto-parietal 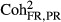 couplings (Fig.5C). Absolute theta spectral coherence was inversely correlated with age both on 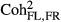 (partial correlation = −0.26), 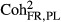 (partial correlation = −0.30) and 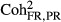 (partial correlation = −0.32) couplings (Fig.5D). Linear regression analysis confirms the inverse correlations between alpha and theta coherence and age (see Tables S3.3. and S3.4., in Appendix S3). As depicted in Fig.5E, baseline-corrected interhemispheric parietal coherence 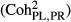 of alpha rhythm recorded during WCST was higher for good than for poor performers and was directly correlated with performance success on WCST (partial correlation = 0.41; Fig.5F). Although no age effects were found on baseline spectral coherences, alpha and theta coherence were higher in baseline than during the performance of the task, in all location pairs. Two-way ANOVA results are presented on Tables S3.1. and S3.2. in Appendix S3.

#### Alpha and theta coupling between frontal left and parietal right scalp regions is directly correlated to performance success on WCST

**Figure 6:**
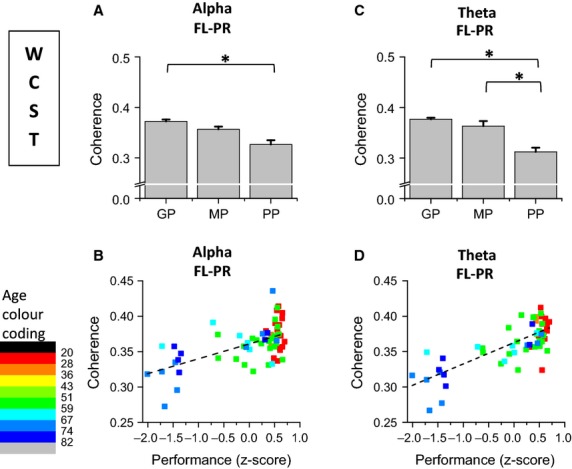
Analysis of performance effects on Spectral Coherence of alpha and theta rhythms recorded during the WCST performance; (A) alpha coherence between FL and PR scalp regions is higher for good than for poor performers; (B) FL-PR coherence of alpha rhythm is directly correlated with *z*-score performance on WCST; (C) Theta coherence between frontal left and parietal right scalp regions is higher for good than for poor performers; FL-PR coherence is higher for medium than for poor performers; (D) Theta coherence between frontal left and parietal right is directly correlated with *z*-score performance on WCST.

Figure6A and C present the performance effects on absolute spectral coherence of alpha and theta rhythms, respectively, for spectral coherence between FL and PR 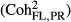 recorded during the WCST. Figure6B and D, present the correlations between alpha coherence and performance *z*-score and between theta coherence and performance *z*-score, respectively.

Plots in Fig.6A show that alpha coherence between frontal left and parietal right scalp regions 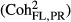 was higher for good than for poor performers and was directly correlated with performance *z*-score on WCST (partial correlation = 0.50) (see Fig.6B). Similarly, theta coherence between frontal left and parietal right scalp regions 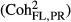 was also higher for good than for poor performers, as plotted in Fig.6C. Theta 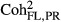 was higher for medium than for poor performers. Theta 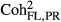 was directly correlated with performance *z*-score on WCST (partial correlation=0.61). The linear dependence between 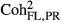 and performance *z*-scores is confirmed through the linear regression analysis presented on Tables S3.3. and S3.4. in Appendix S3.

**Figure 7:**
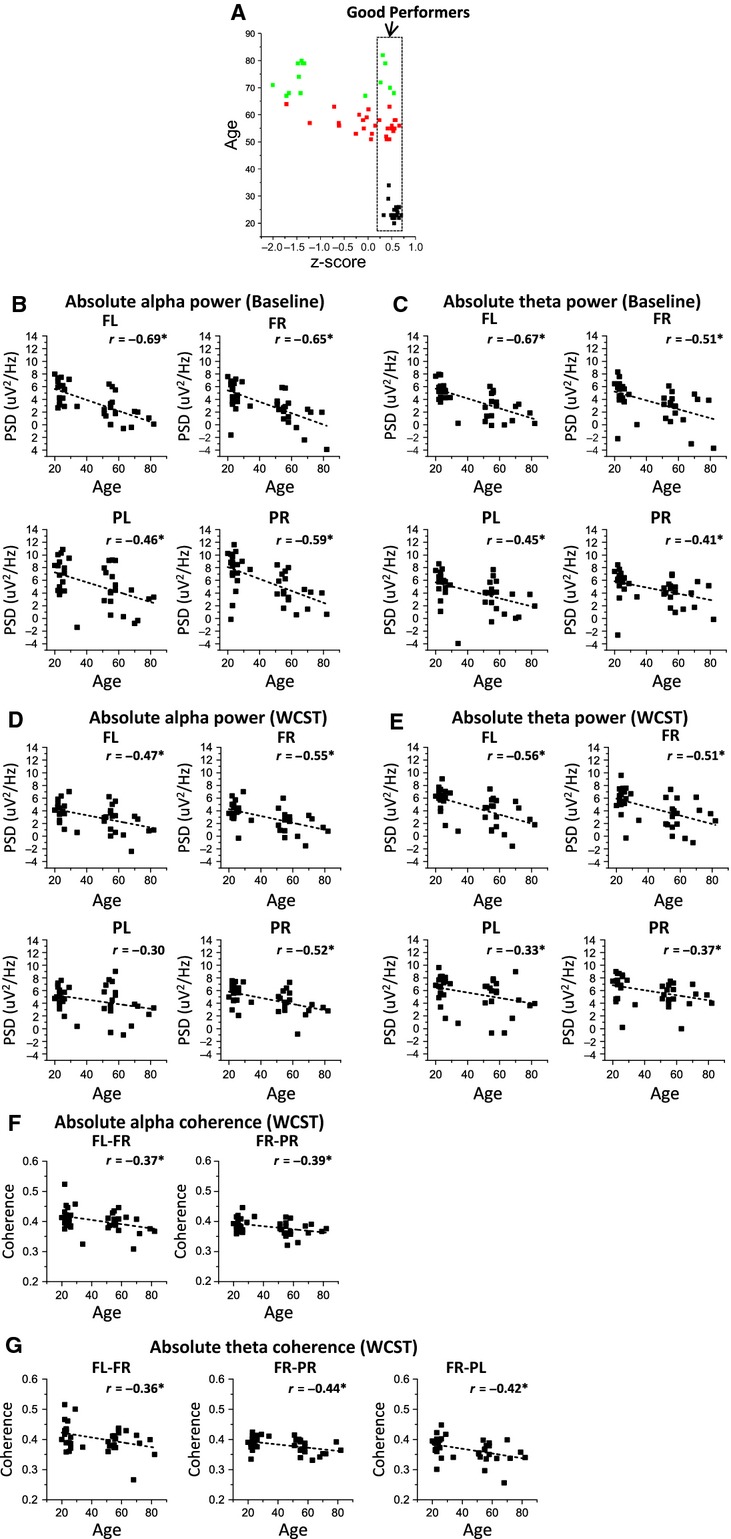
Analysis of age effects on alpha and theta rhythms during baseline and WCST recordings for good performers sub-group; (A) subgroup of good performers *n* = 37; (B) absolute alpha PSD is inversely correlated with age during baseline recordings on good performers; (C) absolute theta PSD is inversely correlated with age during baseline recordings on good performers; (D) absolute alpha PSD is inversely correlated with age during WCST recordings on good performers; (E) absolute theta PSD is inversely correlated with age during WCST recordings on good performers; (F) absolute alpha coherence is inversely correlated with age during WCST recordings on good performers, namely between FL-FR and FR-PR scalp regions; (G) absolute theta coherence is inversely correlated with age during WCST recordings on good performers, namely between FL-FR, FR-PR and FR-PL scalp regions.

**Figure 8:**
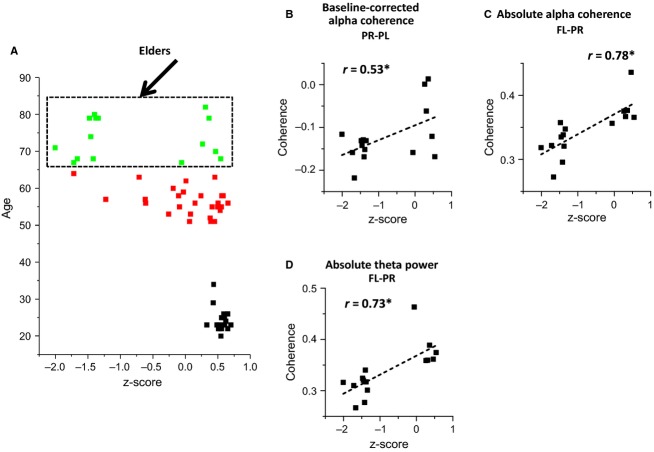
Analysis of performance effects on alpha and theta coherence during WCST and baseline-corrected recordings for elders’ sub-group; (A) subgroup of elderly subjects *n* = 15; (B) Baseline-corrected alpha coherence between PL-PR scalp locations is directly correlated with performance *z*-scores during WCST recordings on elderly subjects; (C) Absolute alpha coherence between FL-PR scalp locations is directly correlated with performance *z*-scores of WCST recordings on elderly subjects; (D) absolute theta coherence between FL-PR scalp locations is directly correlated with performance *z*-scores of WCST recordings on elderly subjects.

In order to confirm the results of sections A (baseline recordings) and B (recordings during the WCST) on participant sub-populations affected by only one factor (i.e. age or performance), the age effects on PSD and spectral coherence were also analyzed on a sub-group of subjects with good performance (*z*-score = 0.51 ± 0.11 SD) on WCST (Fig.7) and performance effects on PSD and coherence were studied on a sub-group consisting of elderly subjects (age = 73.6 ± 5.6 SD) only (Fig.8). Figure7B and C confirm an inverse correlation of both alpha and theta baseline PSD with age, when only considering good performers. Figure7D and E confirm similar correlations during the WCST. Coherence decrease with age was also seen for the subgroup of subjects with good performance on WCST (see Fig.7F and G), for the same region pairs identified on all-subject analysis (Fig.5). Direct correlation between coherence and performance but not with age was also observed when only considering elderly subjects, both for alpha 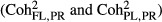 and theta 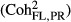 rhythms, as shown in Fig.8B, C and D.

### Alpha peak frequency

#### Frequency of alpha peak recorded during WCST performance is inversely correlated with age on left hemisphere

**Figure 9:**
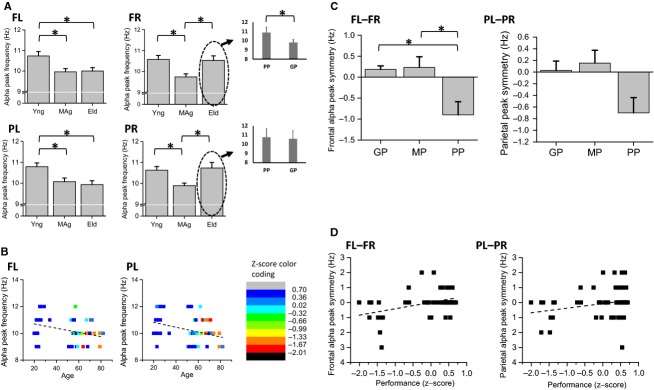
Analysis of age and performance effects on alpha peak frequency (APF) acquired simultaneously to WCST performance; (A) APF is higher for young than middle-age subjects overall four electrode pools and higher than APF of elders on FL and PL electrode pools; APF is higher for elders than middle-age subjects on FR and PR scalp regions; (B) APF is inversely correlated with age on FL and PL electrode pools; (C) difference between left and right APF is higher for good and medium performers than for poor performers on frontal scalp regions; (D) difference between left and right APF is directly correlated with performance *z*-scores on frontal and parietal scalp regions.

Figure9A present the alpha peak frequency (APF) for FL, FR, PL and PR scalp regions and Fig.9B shows the correlation between APF and age for FL and PL scalp regions.

APF recorded from young subjects during the WCST was higher than the one found in mid-age subjects on FL, FR, PL, and PR electrode pools and higher than elders’ APF on FL and PL electrode pools. APF was inversely correlated with age on FL (partial correlation = −0.28) and PL (partial correlation = −0.40) electrode pools (Fig.9B). Although elders’ APF was higher than midage subjects’ on FR and PR scalp regions (Fig.9A), a more detailed analysis of the elders’ APF on FR sites reveals that elders with good performance at the WCST present an alpha frequency slowing as measured on the left hemisphere (inset plot of Fig.9A). Both linear regression analysis and two-way ANOVA statistical designs confirm the inverse correlation between APF and age on the left hemisphere (see Tables S5.1–S5.4., in Appendix S5).

#### Lateral asymmetry of alpha peak frequency is correlated with performance success on WCST

Figure9C presents the lateral asymmetry of APF for frontal and parietal scalp regions and Fig.9D shows the correlations between APF symmetry and performance *z*-scores for frontal (difference between FL and FR APFs) and parietal (difference between PL and PR APFs) APF asymmetries. The frontal difference between left and right (lateral asymmetry) APF was higher for good and medium performers than for poor performers, the latter being mostly elders. Both frontal and parietal lateral asymmetries were directly correlated with performance *z*-scores (FL-FR: partial correlation = 0.41; PL-PR: partial correlation = 0.26). Linear regression analysis and two-way ANOVA statistical designs confirm the direct correlation between alpha peak symmetry and *z*-scores on frontal sites (see details in section Appendix S5 of supplementary data).

## Discussion

The main goal of this study was to identify the effects of age on EEG markers in a task testing working memory and rule shifting. In baseline recordings (i.e., resting state), the power spectral density of alpha and theta rhythms from older subjects were globally reduced in respect to younger ones. Similar reductions were observed while subjects were performing the WCST. Considering partial correlations and controlling for the co-linearity between age and performance *z*-scores, alpha power was inversely correlated with subject age only on the right hemisphere while theta power was inversely correlated with age on frontal locations. Some aging studies, especially on Alzheimer’s disease, have been showing that a decrease in frontal alpha power is a marker for cognitive aging (Jeong 2004), specifically reporting that alpha power decreases with age. Alpha power seems to be associated with the inhibition of irrelevant information, allowing the maintenance of focus and concentration on the task in hands (Werkle-Bergner et al. 2012). The inhibition of irrelevant information is also important for the working memory processes, since working memory relates to the ability to maintain and manipulate information in memory for a short period of time (Werkle-Bergner et al. 2012). Functional imaging studies with simultaneous EEG acquisition have shown that alpha activity is directly correlated with activity in default and self-referential networks and negatively correlated with activity in attention networks (Laufs et al. 2003; Mantini et al. 2007). In the results herein presented, the negative difference between task and baseline-recorded alpha power, across all subjects regardless of age, may indeed represent the engagement of the subject in the task and thus reflect the assignment to attentional mechanisms. In contrast, the increase in task-recorded theta power in respect to the baseline period may reflect the efficient recruitment of working memory processes. This result is expected since the subject is not engaged in any cognitive task during the baseline period (black screen) thus, not recruiting working memory processes that usually involve theta oscillations in its encoding phase (Cummins and Finnigan 2007; Nyhus and Curran 2010). Particularly on parietal left scalp region, baseline-corrected theta power does not correlate with age but is directly correlated with performance *z*-scores instead. This may reflect influences from theta activity of the medial temporal lobe and hippocampus on memory encoding and retrieval (Guderian et al. 2009). Interestingly, low-frequency EEG rhythms with no baseline correction may apparently be regarded as markers of the aging brain, since their spectral power allows mainly the discrimination between young and older subjects. In contrast, baseline-corrected theta power on parietal regions seems a rather promising marker for performance prediction independent of age factor.

Regarding spectral coherence, while frontal interhemispheric (alpha and theta 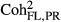) and fronto-parietal (alpha and theta 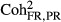; theta 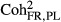) spectral coherences seem to be generally reduced as a function of age, the specific baseline-corrected alpha parietal 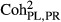 and absolute alpha and theta fronto-parietal 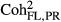 coherences seem sensitive to performance success on the WCST. Our findings suggest that scalp-wise coherence on theta and alpha rhythms are progressively degraded with age. In particular, coherence on frontal and fronto-parietal regions, which are often associated with working memory processes (Sarnthein et al. 1998; Sauseng et al. 2005), executive functions (Sauseng et al. 2005), and attention (Carrillo-de-la-Peña and García-Larrea 2007; Holz et al. 2010; Werkle-Bergner et al. 2012), seem to be particularly sensitive to the aging effect. High coherence is generally associated with augmented linear functional connections and information transfer, which is crucial to a correct use of working memory (Jiang 2005; Rossini et al. 2007), namely in the theta band (Klimesch 1999; González-Hernández et al. 2002). These results are in agreement with other studies that have shown a decline of coherence with age and associated deficits in cognitive function (Jiang 2005; Werkle-Bergner et al. 2012). The overall higher alpha coherence seen during baseline recordings seems to reflect an idling state of several brain regions which are later attenuated by task engagement. Although comparable results were seen to the theta rhythm, the same mechanism may not explain them.

In our results, the alpha peak frequency seems to decrease with age on the left hemisphere. Evidence can be seen from the negative correlations between alpha peak frequency and age, in the left frontal and parietal regions. Alpha peak frequency has been likewise directly correlated with working memory capacity and attention (Klimesch 1999). However, the herein presented results show an apparent age modulation of the alpha peak frequency uniquely for the left hemisphere. On the right hemisphere, poor performers, whom are mostly elders, present an alpha peak frequency as high as the younger subjects’, while the elders with good performance also present an alpha frequency slowing on the right hemisphere. Accordingly, the peak difference between left and right hemispheres reveals an asymmetry for subjects with poor performance on WCST, which may account for the lack of frontal coherence in elders (Fig.5A), and may therefore be regarded as a correlate of cognitive deficit in elders. The right frontal alpha of cognitively diminished elders seems to equal the frequencies measured on young subjects. Although the alpha peak covariance with age requires further study, the herein presented results suggest a generalized alpha peak frequency slowing with aging, while a topographic reorganization of the alpha rhythm source generators might be associated with cognitive impairment at a later age.

From the results presented herein, the correct functioning of frontal brain regions, and specifically the prefrontal cortex, seems critical for a good performance in the WCST (Barceló 2001; Sumitani et al. 2006). However, the parietal regions are also essential for the correct recruitment of attention, working memory and visual processing mechanisms. Thus our results corroborate the idea that the WCST is not a test that assesses only the functioning of frontal brain regions (Monchi et al. 2001; Wang et al. 2001; González-Hernández et al. 2002). The communication between the frontal and temporal, parietal and occipital areas is known to support an efficient performance in the WCST (Holz et al. 2010; Kawasaki et al. 2014). Specifically, interhemispheric long-range correlates such as alpha and theta FL-PR coherence and baseline-corrected parietal alpha coherence are crucial for the correct judgment of stimuli categorization and memory-matching, as it seems to be the case on visual working memory tasks (Knecht et al. 2000).

In summary, the results herein presented demonstrate that both alpha and theta power, as well as coherence, play important roles in WCST performance and are influenced by aging. On one hand, high level of theta power and coherence seems to be a requisite for efficient use of memory resources as shown by the performance on WCST. On other hand, high level of alpha power and coherence seems necessary for correct management of attentional mechanisms during the performance of the WCST. To the best of our knowledge, the performance-specific EEG correlates such as alpha and theta coherence between FL and PR, baseline-corrected theta power on PL and parietal alpha coherence have not been previously reported and therefore are herein suggested as potential age-independent EEG correlates of cognitive performance. Besides leading to a better understanding of the age effects on cognitive performance, the present results also contribute to the identification of EEG markers of cognitive performance that can be employed in the development of new cognitive intervention protocols (e.g., neurofeedback) for the amelioration of cognitive deficits and enhancement of executive functions.

## Supporting Information

**Appendix S1.** Statistic tables of the age effects on power spectral density during baseline recordings. **Appendix S2.** Statistic tables of the age effects on power spectral density during WCST performance. **Appendix S3.** Statistic tables of the age effects on spectral coherence during WCST performance. **Appendix S4.** Statistic tables of performance effects on baseline-corrected spectral coherence during WCST performance. **Appendix S5.** Statistic tables of age and performance effects on Alpha Peak Frequency (APF) during WCST performance.
