# Supplementary material for: Age effects on EEG correlates of the Wisconsin Card Sorting Test
Source: Physiol Rep. 2015 Jul 26;3(7):e12390. doi: 10.14814/phy2.12390 (PMC4552514; doi:10.14814/phy2.12390)
Supplement: Supplementary file 1 [file phy20003-e12390-sd1.docx]

**Supplementary Data**

1. **Statistic tables of the age effects on power spectral density during baseline recordings**

Table I.1. Two-way ANOVA of alpha power spectral density during baseline recordings (* identifies statistical significant differences at 95% confidence with Bonferroni corrections).

| Dependent Variable | | Independent Variables | | Mean Difference (I-J) | SEM | p-value |
| --- | --- | --- | --- | --- | --- | --- |
|  |  | Age Cluster (I) | Age Cluster (J) |  |  |  |
| **Alpha Power** | **FL** | MAg | Eld | 0.37 | 0.67 | 1.00 |
|  |  | Yng | Eld | 2.81* | 0.72 | 0.00 |
|  |  |  | MAg | 2.44* | 0.62 | 0.00 |
|  | **FR** | MAg | Eld | 0.17 | 0.71 | 1.00 |
|  |  | Yng | Eld | 2.42* | 0.76 | 0.01 |
|  |  |  | MAg | 2.25* | 0.66 | 0.00 |
|  | **PL** | MAg | Eld | 1.26 | 0.98 | 0.61 |
|  |  | Yng | Eld | 2.68* | 1.05 | 0.04 |
|  |  |  | MAg | 1.42 | 0.91 | 0.37 |
|  | **PR** | MAg | Eld | 0.66 | 0.89 | 1.00 |
|  |  | Yng | Eld | 2.98* | 0.96 | 0.01 |
|  |  |  | MAg | 2.32* | 0.83 | 0.02 |

Table I.2. Two-way ANOVA of theta power spectral density during baseline recordings (* identifies statistical significant differences at 95% confidence with Bonferroni corrections).

| Dependent Variable | | Independent Variables | | Mean Difference (I-J) | SEM | p-value |
| --- | --- | --- | --- | --- | --- | --- |
|  |  | Age Cluster (I) | Age Cluster (J) |  |  |  |
| **Theta Power** | **FL** | MAg | Eld | 1.01 | 0.62 | 0.33 |
|  |  | Yng | Eld | 3.37* | 0.67 | 0.00 |
|  |  |  | MAg | 2.35* | 0.58 | 0.00 |
|  | **FR** | MAg | Eld | 1.49 | 0.78 | 0.19 |
|  |  | Yng | Eld | 3.01* | 0.84 | 0.00 |
|  |  |  | MAg | 1.53 | 0.73 | 0.12 |
|  | **PL** | MAg | Eld | 1.54 | 0.82 | 0.19 |
|  |  | Yng | Eld | 2.97* | 0.87 | 0.00 |
|  |  |  | MAg | 1.42 | 0.76 | 0.20 |
|  | **PR** | MAg | Eld | 1.64 | 0.71 | 0.08 |
|  |  | Yng | Eld | 2.76* | 0.77 | 0.00 |
|  |  |  | MAg | 1.12 | 0.66 | 0.29 |

Table I.3. Linear regression analysis of the age effects on alpha power spectral density during baseline recordings (* identifies statistical significant correlations at 95% confidence).

| Independent Variable | | Dependent Variable | Standardized Coefficients | t statistic | p-value | Correlations | |
| --- | --- | --- | --- | --- | --- | --- | --- |
|  |  |  |  |  |  | Total | Partial |
| **Alpha Power** | **FL** | Age | -0.52 | -3.60 | 0.00 | -0.49 | -0.43* |
|  |  | zscore | -0.05 | -0.37 | 0.71 | 0.27 | -0.05 |
|  | **FR** | Age | -0.46 | -3.07 | 0.00 | -0.43 | -0.37* |
|  |  | zscore | -0.05 | -0.33 | 0.75 | 0.23 | -0.04 |
|  | **PL** | Age | -0.37 | -2.38 | 0.02 | -0.33 | -0.30* |
|  |  | zscore | -0.07 | -0.43 | 0.67 | 0.16 | -0.06 |
|  | **PR** | Age | -0.49 | -3.25 | 0.00 | -0.41 | -0.39* |
|  |  | zscore | -0.13 | -0.88 | 0.38 | 0.17 | -0.11 |

Table I.4. Linear regression analysis of the age effects on theta power spectral density during baseline recordings (* identifies statistical significant correlations at 95% confidence).

| Independent Variable | | Dependent Variable | Standardized Coefficients | t statistic | p-value | Correlations | |
| --- | --- | --- | --- | --- | --- | --- | --- |
|  |  |  |  |  |  | Total | Partial |
| **Theta Power** | **FL** | Age | -0.68 | -5.18 | 0.00 | -0.61 | -0.56* |
|  |  | zscore | -0.11 | -0.82 | 0.41 | 0.31 | -0.11 |
|  | **FR** | Age | -0.53 | -3.58 | 0.00 | -0.46 | -0.43* |
|  |  | zscore | -0.12 | -0.79 | 0.44 | 0.21 | -0.10 |
|  | **PL** | Age | -0.45 | -3.02 | 0.00 | -0.45 | -0.37* |
|  |  | zscore | -0.01 | -0.04 | 0.97 | 0.27 | -0.01 |
|  | **PR** | Age | -0.39 | -2.62 | 0.01 | -0.43 | -0.33* |
|  |  | zscore | 0.07 | 0.44 | 0.66 | 0.31 | 0.06 |

1. **Statistic tables of the age effects on power spectral density during WCST performance.**

Table II.1. Two-way ANOVA of alpha power spectral density during WCST performance (* identifies statistical significant differences at 95% confidence with Bonferroni corrections).

| Dependent Variable | | Independent Variables | | Mean Difference (I-J) | SEM | p-value |
| --- | --- | --- | --- | --- | --- | --- |
|  |  | Age Cluster (I) | Age Cluster (J) |  |  |  |
| **Alpha Power** | **FL** | MAg | Eld | 0.88 | 0.65 | 0.54 |
|  |  | Yng | Eld | 2.04* | 0.70 | 0.01 |
|  |  |  | MAg | 1.16 | 0.60 | 0.18 |
|  | **FR** | MAg | Eld | 0.33 | 0.60 | 1.00 |
|  |  | Yng | Eld | 2.23* | 0.64 | 0.00 |
|  |  |  | MAg | 1.90* | 0.55 | 0.00 |
|  | **PL** | MAg | Eld | 1.53 | 0.86 | 0.24 |
|  |  | Yng | Eld | 2.08 | 0.92 | 0.08 |
|  |  |  | MAg | 0.55 | 0.80 | 1.00 |
|  | **PR** | MAg | Eld | 1.11 | 0.72 | 0.40 |
|  |  | Yng | Eld | 2.25* | 0.78 | 0.02 |
|  |  |  | MAg | 1.14 | 0.67 | 0.29 |

Table II.2. Two-way ANOVA of theta power spectral density during WCST performance (* identifies statistical significant differences at 95% confidence with Bonferroni corrections).

| Dependent Variable | | Independent Variables | | Mean Difference (I-J) | SEM | p-value |
| --- | --- | --- | --- | --- | --- | --- |
|  |  | Age Cluster (I) | Age Cluster (J) |  |  |  |
| **Theta Power** | **FL** | MAg | Eld | 1.51 | 0.74 | 0.14 |
|  |  | Yng | Eld | 3.71* | 0.79 | 0.00 |
|  |  |  | MAg | 2.20* | 0.69 | 0.01 |
|  | **FR** | MAg | Eld | 1.11 | 0.73 | 0.41 |
|  |  | Yng | Eld | 3.64* | 0.79 | 0.00 |
|  |  |  | MAg | 2.53* | 0.68 | 0.00 |
|  | **PL** | MAg | Eld | 2.09 | 0.88 | 0.06 |
|  |  | Yng | Eld | 3.55* | 0.94 | 0.00 |
|  |  |  | MAg | 1.46 | 0.82 | 0.24 |
|  | **PR** | MAg | Eld | 2.16* | 0.79 | 0.03 |
|  |  | Yng | Eld | 3.48* | 0.85 | 0.00 |
|  |  |  | MAg | 1.32 | 0.74 | 0.24 |

Table II.3. Linear regression analysis of the age effects on alpha power spectral density during WCST performance (* identifies statistical significant correlations at 95% confidence).

| Independent Variable | | Dependent Variable | Standardized Coefficients | t statistic | p-value | Correlations | |
| --- | --- | --- | --- | --- | --- | --- | --- |
|  |  |  |  |  |  | Total | Partial |
| **Alpha Power** | **FL** | Age | -0.21 | -1.40 | 0.17 | -0.37 | -0.18 |
|  |  | zscore | 0.26 | 1.70 | 0.09 | 0.39 | 0.22 |
|  | **FR** | Age | -0.37 | -2.51 | 0.01 | -0.46 | -0.31* |
|  |  | zscore | 0.14 | 0.95 | 0.35 | 0.37 | 0.12 |
|  | **PL** | Age | -0.14 | -0.87 | 0.39 | -0.28 | -0.11 |
|  |  | zscore | 0.23 | 1.44 | 0.16 | 0.31 | 0.19 |
|  | **PR** | Age | -0.36 | -2.34 | 0.02 | -0.36 | -0.29* |
|  |  | zscore | 0.00 | -0.02 | 0.98 | 0.22 | 0.00 |

Table II.4. Linear regression analysis of the age effects on theta power spectral density during WCST performance (* identifies statistical significant correlations at 95% confidence).

| Independent Variable | | Dependent Variable | Standardized Coefficients | t statistic | p-value | Correlations | |
| --- | --- | --- | --- | --- | --- | --- | --- |
|  |  |  |  |  |  | Total | Partial |
| **Theta Power** | **FL** | Age | -0.51 | -3.66 | 0.00 | -0.54 | -0.43* |
|  |  | zscore | 0.05 | 0.38 | 0.71 | 0.37 | 0.05 |
|  | **FR** | Age | -0.48 | -3.41 | 0.00 | -0.53 | -0.41* |
|  |  | zscore | 0.08 | 0.59 | 0.56 | 0.38 | 0.08 |
|  | **PL** | Age | -0.28 | -1.96 | 0.05 | -0.45 | -0.25* |
|  |  | zscore | 0.27 | 1.89 | 0.06 | 0.45 | 0.24 |
|  | **PR** | Age | -0.28 | -1.96 | 0.05 | -0.46 | -0.25* |
|  |  | zscore | 0.29 | 2.01 | 0.05 | 0.46 | 0.25* |

1. **Statistic tables of the age effects on spectral coherence during WCST performance.**

Table III.1. Two-way ANOVA of alpha spectral coherence during WCST performance (* identifies statistical significant differences at 95% confidence with Bonferroni corrections).

| Dependent Variable | | Independent Variables | | Mean Difference (I-J) | SEM | p-value |
| --- | --- | --- | --- | --- | --- | --- |
|  |  | Age Cluster (I) | Age Cluster (J) |  |  |  |
| **Alpha Spectral Coherence** | **FL-FR** | MAg | Eld | 0.027* | 0.010 | 0.03 |
|  |  | Yng | Eld | 0.051* | 0.011 | 0.00 |
|  |  |  | MAg | 0.024* | 0.009 | 0.04 |
|  | **FL-PL** | MAg | Eld | 0.016 | 0.011 | 0.48 |
|  |  | Yng | Eld | 0.019 | 0.012 | 0.40 |
|  |  |  | MAg | 0.002 | 0.011 | 1.00 |
|  | **FL-PR** | MAg | Eld | 0.015 | 0.008 | 0.20 |
|  |  | Yng | Eld | 0.029* | 0.009 | 0.01 |
|  |  |  | MAg | 0.013 | 0.008 | 0.27 |
|  | **FR-PL** | MAg | Eld | 0.005 | 0.009 | 1.00 |
|  |  | Yng | Eld | 0.028* | 0.010 | 0.02 |
|  |  |  | MAg | 0.023* | 0.008 | 0.02 |
|  | **FR-PR** | MAg | Eld | 0.001 | 0.008 | 1.00 |
|  |  | Yng | Eld | 0.024* | 0.009 | 0.03 |
|  |  |  | MAg | 0.023* | 0.008 | 0.01 |
|  | **PL-PR** | MAg | Eld | 0.003 | 0.011 | 1.00 |
|  |  | Yng | Eld | 0.025 | 0.011 | 0.09 |
|  |  |  | MAg | 0.022 | 0.010 | 0.08 |

Table III.2. Two-way ANOVA of theta spectral coherence during WCST performance (* identifies statistical significant differences at 95% confidence with Bonferroni corrections).

| Dependent Variable | | Independent Variables | | Mean Difference (I-J) | SEM | p-value |
| --- | --- | --- | --- | --- | --- | --- |
|  |  | Age Cluster (I) | Age Cluster (J) |  |  |  |
| **Theta Spectral Coherence** | **FL-FR** | MAg | Eld | 0.028 | 0.013 | 0.09 |
|  |  | Yng | Eld | 0.056* | 0.014 | 0.00 |
|  |  |  | MAg | 0.027 | 0.012 | 0.07 |
|  | **FL-PL** | MAg | Eld | 0.019 | 0.011 | 0.25 |
|  |  | Yng | Eld | 0.022 | 0.011 | 0.17 |
|  |  |  | MAg | 0.003 | 0.010 | 1.00 |
|  | **FL-PR** | MAg | Eld | 0.026* | 0.008 | 0.00 |
|  |  | Yng | Eld | 0.042* | 0.008 | 0.00 |
|  |  |  | MAg | 0.016 | 0.007 | 0.08 |
|  | **FR-PL** | MAg | Eld | 0.022 | 0.011 | 0.16 |
|  |  | Yng | Eld | 0.053* | 0.012 | 0.00 |
|  |  |  | MAg | 0.031* | 0.010 | 0.01 |
|  | **FR-PR** | MAg | Eld | 0.013 | 0.008 | 0.31 |
|  |  | Yng | Eld | 0.034* | 0.009 | 0.00 |
|  |  |  | MAg | 0.020* | 0.008 | 0.03 |
|  | **PL-PR** | MAg | Eld | 0.013 | 0.012 | 0.90 |
|  |  | Yng | Eld | 0.027 | 0.013 | 0.14 |
|  |  |  | MAg | 0.014 | 0.011 | 0.69 |

Table III.3. Linear regression analysis of the age effects on alpha spectral coherence during WCST performance (* identifies statistical significant correlations at 95% confidence).

| Independent Variable | | Dependent Variable | Standardized Coefficients | t statistic | p-value | Correlations | |
| --- | --- | --- | --- | --- | --- | --- | --- |
|  |  |  |  |  |  | Total | Partial |
| **Alpha Spectral Coherence** | **FL-FR** | Age | -0.32 | -2.27 | 0.03 | -0.48 | -0.29* |
|  |  | zscore | 0.27 | 1.87 | 0.07 | 0.46 | 0.24 |
|  | **FL-PL** | Age | -0.03 | -0.20 | 0.85 | -0.18 | -0.03 |
|  |  | zscore | 0.24 | 1.50 | 0.14 | 0.26 | 0.19 |
|  | **FL-PR** | Age | 0.03 | 0.20 | 0.84 | -0.34 | 0.03 |
|  |  | zscore | 0.59 | 4.35 | 0.00 | 0.58 | 0.50* |
|  | **FR-PL** | Age | -0.30 | -1.92 | 0.06 | -0.36 | -0.24 |
|  |  | zscore | 0.10 | 0.63 | 0.53 | 0.28 | 0.08 |
|  | **FR-PR** | Age | -0.33 | -2.16 | 0.04 | -0.37 | -0.27* |
|  |  | zscore | 0.06 | 0.39 | 0.69 | 0.27 | 0.05 |
|  | **PL-PR** | Age | -0.12 | -0.75 | 0.46 | -0.28 | -0.10 |
|  |  | zscore | 0.27 | 1.71 | 0.09 | 0.34 | 0.22 |

Table III.4. Linear regression analysis of the age effects on theta spectral coherence during WCST performance (* identifies statistical significant correlations at 95% confidence).

| Independent Variable | | Dependent Variable | Standardized Coefficients | t statistic | p-value | Correlations | |
| --- | --- | --- | --- | --- | --- | --- | --- |
|  |  |  |  |  |  | Total | Partial |
| **Theta Spectral Coherence** | **FL-FR** | Age | -0.30 | -2.03 | 0.05 | -0.43 | -0.26* |
|  |  | zscore | 0.22 | 1.46 | 0.15 | 0.40 | 0.19 |
|  | **FL-PL** | Age | -0.16 | -1.02 | 0.31 | -0.25 | -0.13 |
|  |  | zscore | 0.15 | 0.91 | 0.37 | 0.25 | 0.12 |
|  | **FL-PR** | Age | -0.01 | -0.08 | 0.93 | -0.44 | -0.01 |
|  |  | zscore | 0.70 | 5.86 | 0.00 | 0.70 | 0.61* |
|  | **FR-PL** | Age | -0.34 | -2.41 | 0.02 | -0.49 | -0.30* |
|  |  | zscore | 0.23 | 1.61 | 0.11 | 0.44 | 0.21 |
|  | **FR-PR** | Age | -0.39 | -2.59 | 0.01 | -0.44 | -0.32* |
|  |  | zscore | 0.09 | 0.61 | 0.54 | 0.33 | 0.08 |
|  | **PL-PR** | Age | -0.04 | -0.23 | 0.82 | -0.24 | -0.03 |
|  |  | zscore | 0.33 | 2.11 | 0.04 | 0.35 | 0.27* |

1. **Statistic tables of performance effects on baseline-corrected spectral coherence during WCST performance.**

Table IV.1. Two-way ANOVA of baseline-corrected alpha and theta spectral coherence and theta power spectral density during WCST performance (* identifies statistical significant differences at 95% confidence with Bonferroni corrections).

| Dependent Variable | | Independent Variables | | Mean Difference (I-J) | SEM | p-value |
| --- | --- | --- | --- | --- | --- | --- |
|  |  | Age Cluster (I) | Age Cluster (J) |  |  |  |
| **Alpha Coherence** | **PL-PR** | MP | PP | 0.01 | 0.02 | 1.00 |
|  |  | GP | PP | 0.05 | 0.02 | 0.01* |
|  |  | GP | MP | 0.04 | 0.01 | 0.06 |
|  | **FL-PR** | MP | PP | 0.02 | 0.01 | 0.11 |
|  |  | GP | PP | 0.04 | 0.01 | 0.00* |
|  |  | GP | MP | 0.01 | 0.01 | 0.31 |
| **Theta Coherence** | **FL-PR** | MP | PP | 0.04 | 0.01 | 0.00* |
|  |  | GP | PP | 0.05 | 0.01 | 0.00* |
|  |  | GP | MP | 0.01 | 0.01 | 0.44 |
| **Theta Power** | **PL** | MP | PP | 1.83 | 0.84 | 0.10 |
|  |  | GP | PP | 2.40 | 0.71 | 0.00* |
|  |  | GP | MP | 0.56 | 0.64 | 1.00 |

Table IV.2. Linear regression analysis of the performance effects on baseline-corrected theta power spectral density and alpha spectral coherence during WCST performance (* identifies statistical significant correlations at 95% confidence).

| Independent Variable | | Dependent Variable | Standardized Coefficients | t statistic | p-value | Correlations | |
| --- | --- | --- | --- | --- | --- | --- | --- |
|  |  |  |  |  |  | Total | Partial |
| **Theta Power** | **PL** | Age | 0.17 | 1.08 | 0.29 | -0.07 | 0.14 |
|  |  | zscore | 0.39 | 2.48 | 0.02 | 0.29 | 0.31* |
| **Alpha Coherence** | **PL-PR** | Age | 0.32 | 2.09 | 0.04 | -0.01 | 0.26* |
|  |  | zscore | 0.52 | 3.46 | 0.00 | 0.33 | 0.41* |

1. **Statistic tables of age and performance effects on Alpha Peak Frequency (APF) during WCST performance**

Table V.1. Two-way ANOVA of alpha peak frequency during WCST performance (* identifies statistical significant differences at 95% confidence with Bonferroni corrections).

| Dependent Variable | | Independent Variables | | Mean Difference (I-J) | SEM | p-value |
| --- | --- | --- | --- | --- | --- | --- |
|  |  | Age Cluster (I) | Age Cluster (J) |  |  |  |
| **Alpha Peak Frequency** | **FL** | MAg | Eld | -0.04 | 0.26 | 1.00 |
|  |  |  | Yng | -0.77* | 0.24 | 0.01 |
|  |  | Yng | Eld | 0.74* | 0.28 | 0.04 |
|  |  |  | MAg | 0.77* | 0.24 | 0.01 |
|  | **FR** | MAg | Eld | -0.79* | 0.25 | 0.01 |
|  |  |  | Yng | -0.84* | 0.23 | 0.00 |
|  |  | Yng | Eld | 0.05 | 0.27 | 1.00 |
|  |  |  | MAg | 0.84* | 0.23 | 0.00 |
|  | **PL** | MAg | Eld | 0.14 | 0.27 | 1.00 |
|  |  |  | Yng | -0.72* | 0.25 | 0.02 |
|  |  | Yng | Eld | 0.86* | 0.29 | 0.01 |
|  |  |  | MAg | 0.72* | 0.25 | 0.02 |
|  | **PR** | MAg | Eld | -0.84* | 0.26 | 0.01 |
|  |  |  | Yng | -0.74* | 0.24 | 0.01 |
|  |  | Yng | Eld | -0.10 | 0.28 | 1.00 |
|  |  |  | MAg | 0.74* | 0.24 | 0.01 |

Table V.2. Two-way ANOVA of alpha peak symmetry during WCST performance (* identifies statistical significant differences at 95% confidence with Bonferroni corrections).

| Dependent Variable | | Independent Variables | | Mean Difference (I-J) | SEM | p-value |
| --- | --- | --- | --- | --- | --- | --- |
|  |  | Age Cluster (I) | Age Cluster (J) |  |  |  |
| **Alpha Peak Symmetry** | **FL-FR** | MP | PP | 1.13* | 0.30 | 0.00 |
|  |  | GP | PP | 1.08* | 0.26 | 0.00 |
|  |  | GP | MP | -0.05 | 0.23 | 1.00 |
|  | **PL-PR** | MP | PP | 0.85 | 0.38 | 0.09 |
|  |  | GP | PP | 0.73 | 0.32 | 0.08 |
|  |  | GP | MP | -0.13 | 0.29 | 1.00 |

Table V.3. Linear regression analysis of the age and performance effects on alpha peak frequency during WCST performance (* identifies statistical significant correlations at 95% confidence).

| Independent Variable | | Dependent Variable | Standardized Coefficients | t statistic | p-value | Correlations | |
| --- | --- | --- | --- | --- | --- | --- | --- |
|  |  |  |  |  |  | Total | Partial |
| **Alpha Peak Frequency** | **FL** | Age | -0.35 | -2.22 | 0.03 | -0.35 | -0.28* |
|  |  | zscore | 0.01 | 0.06 | 0.95 | 0.22 | 0.01 |
|  | **FR** | Age | -0.37 | -2.40 | 0.02 | -0.10 | -0.30* |
|  |  | zscore | -0.44 | -2.84 | 0.01 | -0.21 | -0.35* |
|  | **PL** | Age | -0.50 | -3.30 | 0.00 | -0.40 | -0.40* |
|  |  | zscore | -0.17 | -1.11 | 0.27 | 0.14 | -0.14 |
|  | **PR** | Age | -0.40 | -2.67 | 0.01 | -0.08 | -0.33* |
|  |  | zscore | -0.53 | -3.49 | 0.00 | -0.28 | -0.42* |

Table V.4. Linear regression analysis of the age and performance effects on alpha peak symmetry during WCST performance (* identifies statistical significant correlations at 95% confidence).

| Independent Variable | | Dependent Variable | Standardized Coefficients | t statistic | p-value | Correlations | |
| --- | --- | --- | --- | --- | --- | --- | --- |
|  |  |  |  |  |  | Total | Partial |
| **Alpha Peak Symmetry** | **FL-FR** | Age | 0.04 | 0.24 | 0.81 | -0.27 | 0.03 |
|  |  | zscore | 0.50 | 3.40 | 0.00 | 0.48 | 0.41* |
|  | **PL-PR** | Age | -0.10 | -0.64 | 0.52 | -0.30 | -0.08 |
|  |  | zscore | 0.32 | 2.09 | 0.04 | 0.38 | 0.26* |
